# Vehicle Detection under Adverse Weather from Roadside LiDAR Data

**DOI:** 10.3390/s20123433

**Published:** 2020-06-17

**Authors:** Jianqing Wu, Hao Xu, Yuan Tian, Rendong Pi, Rui Yue

**Affiliations:** 1 School of Qilu Transportation, Shandong University, Jinan 250061, China; jianqingwusdu@sdu.edu.cn (J.W.); pirendong@mail.sdu.edu.cn (R.P.); 2 Department of Civil and Environmental Engineering, University of Nevada, Reno, NV 89557, USA; haox@unr.edu (H.X.); yuantian@nevada.unr.edu (Y.T.)

**Keywords:** vehicle detection, adverse weather, roadside LiDAR, data processing

## Abstract

Roadside light detection and ranging (LiDAR) is an emerging traffic data collection device and has recently been deployed in different transportation areas. The current data processing algorithms for roadside LiDAR are usually developed assuming normal weather conditions. Adverse weather conditions, such as windy and snowy conditions, could be challenges for data processing. This paper examines the performance of the state-of-the-art data processing algorithms developed for roadside LiDAR under adverse weather and then composed an improved background filtering and object clustering method in order to process the roadside LiDAR data, which was proven to perform better under windy and snowy weather. The testing results showed that the accuracy of the background filtering and point clustering was greatly improved compared to the state-of-the-art methods. With this new approach, vehicles can be identified with relatively high accuracy under windy and snowy weather.

## 1. Introduction

Adverse weather can negatively influence transportation performance in two aspects: decreasing the operational efficiency and increasing the crash risk. Fortunately, as connected vehicle (CV) technology becomes more realistic, the overall operational efficiency and traffic safety can greatly benefit from CV technology, especially under adverse weather conditions. However, effectively employing CV technology on the road requires accurate traffic data. The quality of these data could also be influenced by adverse weather, which confuses the judgment of the CV network and causes the loss of operational efficiency and crashes. Therefore, investigating how to improve the accuracy of traffic data under adverse weather is significantly important for current CV technology. Light detection and ranging (LiDAR), an emerging sensor for intelligent transportation systems, has the potential of providing traffic data under good weather conditions [1]. The new 360-degree LiDAR can detect all road users and surrounding environments in a 360-degree horizontal field of view (FOV). Compared to traditional sensors, such as cameras, loop detectors, and radar, LiDAR can work day and night and has higher accuracy for object detection [2]. Airborne and on-board LiDAR (mobile LiDAR) are the traditional installation methods for object detection and remote sensing [3]. Recently, the roadside LiDAR has been a new deployment method for transportation applications. The LiDAR can be installed on a tripod for short-term data collection or on roadside infrastructures (such as a wire pole) for long-term data collection [4,5]. The roadside LiDAR sensor is able to scan the surfaces of all road vehicles (including both connected vehicles and unconnected vehicles) within the detection range by generating 3D point clouds, which provides a perfect solution for filling the data gap of the transition period from unconnected vehicles to connected vehicles [6]. Here, connected vehicles refer to those vehicles that can be engaged in the connected vehicle environment. The high-resolution trajectories of all road users can then be extracted from the roadside LiDAR and can provide valuable information such as driver behavior analysis, fuel consumption, near-crash identification, and prediction [7,8,9,10].

A significant number of studies have been conducted to extract useful traffic information from roadside LiDAR data. The roadside LiDAR data processing procedure typically includes four steps: background filtering, object clustering, object classification, and object tracking [11]. This paper focuses on the first two parts: background filtering and object clustering. The background in roadside LiDAR data usually includes stationary objects such as buildings and the ground surface, and dynamic objects such as waving trees, grasses, and bushes. When referring to stationary objects, the location of the same LiDAR point at different frames is not strictly fixed due to the slight shaking of the LiDAR laser beams [5], which results in difficulties for background filtering. The original method for filtering the background was to search the frames without road users within the detection range [12,13]. However, it may be difficult to select the correct number of frames without any road users at high-volume traffic road segments or intersections. Zhang et al. [14] developed a point association (PA)-based method for background filtering. A frame without any road users was manually selected as a reference frame. Then, a predefined distance threshold was assigned to the background points in the reference frame. Any point with a distance to the roadside LiDAR shorter than the threshold was identified as a background point. However, the threshold needed to be selected based on the users’ experience, which limited the actual application of the PA-based method. Wu et al. [15] developed a point density-based method named 3D density statistic filtering (3D-DSF) for background filtering. The 3D-DSF method does not need to manually select the suitable frames. In their method, the whole detection range is divided into amounts of small cubes, and the point density of each cube in each frame is calculated. Then, by frame aggregation, the sum of the point density over all frames of each cube can be found. A predefined threshold is used to distinguish background cubes from non-background cubes. More details about the 3D-DSF are referred to in [16]. The assumption of this study was that the sum of the point density of the background cube will be much larger than that of the cube with road users. However, a limitation of the 3D-DSF is that it is unable to exclude the background points effectively under congested intersections. Lv et al. [17] developed a raster-based (RA) method using the change in point density as a feature for background filtering. Any cube with a change in point density larger than two in two adjacent frames was considered as background. The testing results showed that the raster-based method could exclude more than 98% of the background points in the three investigated sites. However, all the above-mentioned methods were performed under normal weather. The performance of those background filtering methods under harsh environments, such as strong wind and snow, was not evaluated.

Point clustering means to cluster the points belonging to one object into one group. Zhang et al. [18] used the Euclidean clustering extraction (ECE) algorithm for point clustering. ECE uses two parameters, the cluster size (S) and the tolerance (d), to search the points belonging to one object. Since there are no standard methods for parameter selection, heuristic testing is required to determine the optimal value for different datasets. Wu [5] applied the density-based spatial clustering of applications with noise (DBSCAN) for clustering. The advantage of DBSCAN is that it does not need to know the number of objects in advance. DBSCAN uses epsilon and the minimum number of points to determine whether a point belongs to a group or not. Wu [5] (Wu, 2018) suggested using 1.2 m as epsilon and 10 as the minimum number of points for the input of DBSCAN. Later, Zhao et al. [19] found that the fixed parameters of DBSCAN could not group the points correctly when the object was far away from the LiDAR. The principal reason was that the density of the same object changed with a different distance to the roadside LiDAR. Zhao et al. [19] developed a revised DBSCAN for object clustering based on the distribution feature of the LiDAR point within the space. However, the DBSCAN related algorithms are computationally expensive since they require an extensive search of all points in the point cloud. A previous study [20] also found that the method proposed by Zhao et al. [19] could not cluster the points correctly under snowy weather.

In fact, a large amount of research has been done to process LiDAR data under severe weather conditions [21,22,23,24,25,26,27,28,29]. Wojtanowski et al. [22] found that LiDAR is susceptible to adverse weather conditions. Charron et al. [23] developed a dynamic 3D outlier detection method to remove snow noise from the onboard LiDAR data. The testing results showed that the proposed method could achieve more than 90% precision. Jokela et al. [24] found that LiDAR sensors’ performance decreased with the increasing density of fog and the distance between the target and the LiDAR. The visible range for object detection in the LiDAR relied on the different types of LiDAR. Kutila et al. [25] evaluated the performance of automotive LiDAR in fog and rain. It was found that fog can be a challenge for object detection using the LiDAR at a 905 nm wavelength due to light being scattered by fog particles and a 1550 nm wavelength was recommended to be used in the LiDAR in order to reduce the impact of fog particles. Bijelic et al. [26,27] compared the performance of four different state-of-the-art LiDAR systems. The results showed that all the LiDAR systems decreased in fog and that changing the internal parameters in the LiDAR could improve their functions under adverse weather.

The above-mentioned studies have shown that adverse weather can reduce the resolution of the roadside LiDAR data qualitatively. It is still necessary to quantitatively analyze the influence of different adverse conditions on the roadside LiDAR and to develop new methods that can accommodate background filtering and point clustering for adverse weather conditions.

## 2. Background Filtering

**Figure 1:**
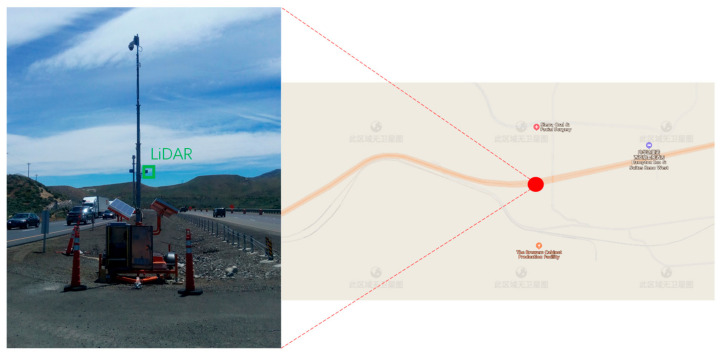
Testing site.

One advantage of roadside LiDAR is that past information (historical frames) can be used to process the current data [30,31]. With this feature, the accuracy of data processing can be greatly improved. In fact, the previously mentioned methods, such as 3D-DSF, RA, and PA, all used historical information to enhance the accuracy of the background filtering. However, for temporary data collection, the wind may influence the resolution of the LiDAR data, especially at windy spots. As a result, non-background points can be misrecognized as background points and background points can be misrecognized as non-background points. For background filtering, 3D-DSF is still the most widely used method for roadside LiDAR data processing [32,33,34,35]. Here, we examined the performance of 3D-DSF under snowy and windy weather conditions. One road segment along the I-80 freeway in Reno was selected as the testing site. The site’s location is shown in Figure 1.

**Figure 2:**
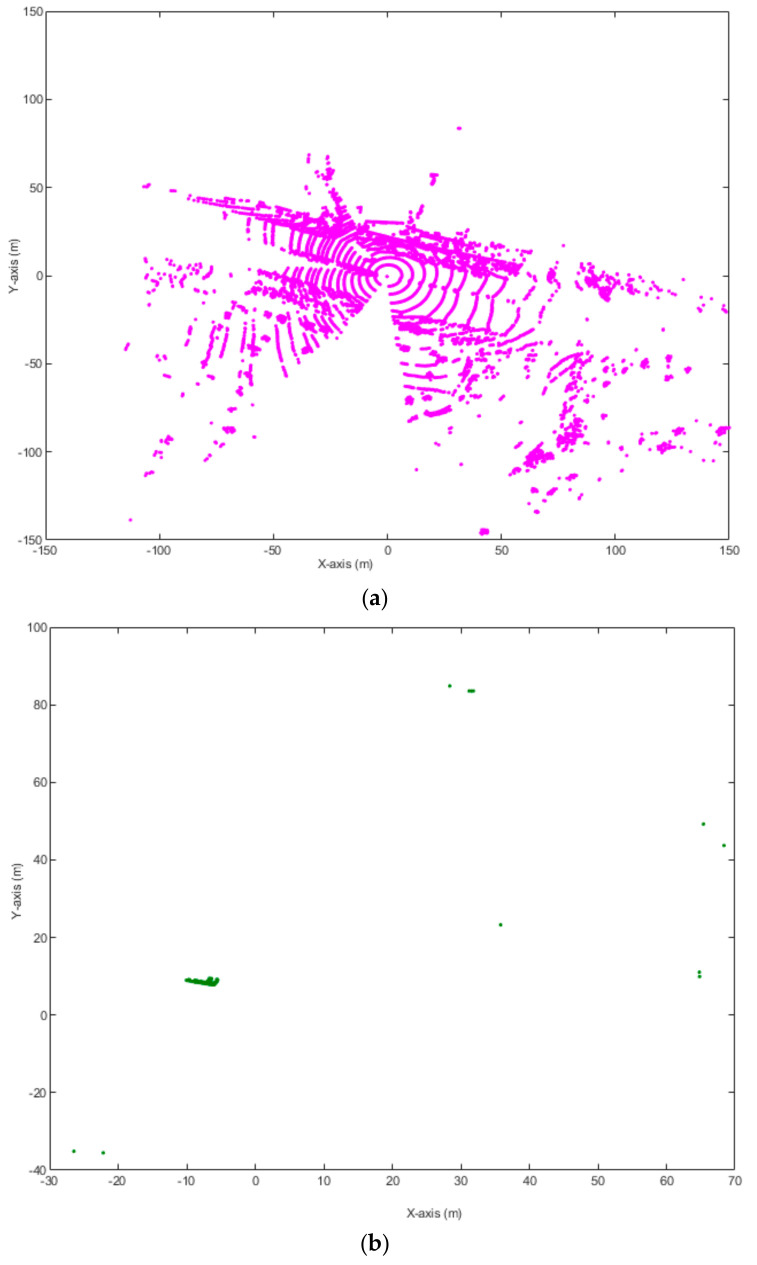

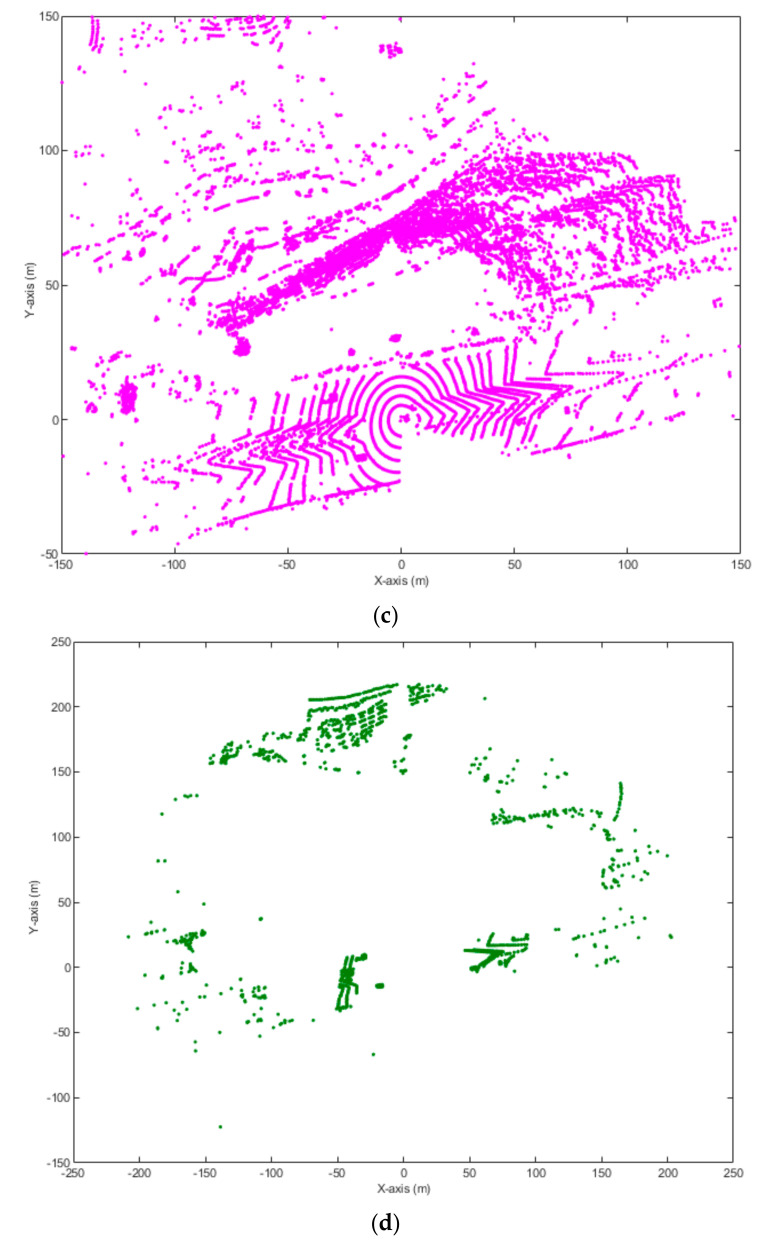
Performance of 3D density statistic filtering (3D-DSF) under windy and non-windy weather: (**a**) No wind before applying 3D-DSF, (**b**) No wind after applying 3D-DSF, (**c**) Strong wind before applying 3D-DSF, (**d**) Strong wind after applying 3D-DSF.

Figure 2 shows an example of 3D-DSF under windy and non-windy weather conditions. Figure 2a,b shows that under normal (non-windy) weather, 3D-DSF can exclude most background points and leave the non-background points in the space. In Figure 2b, we can clearly see where the cluster points are, as they are highlighted in green. Previous research has shown that vehicles can easily be identified after data are applied with 3D-DSF. However, under windy weather, 3D-DSF could not effectively exclude the ground surface, as shown in Figure 2c,d. In Figure 2d, although the background points are partially eliminated, the non-background points and background points are still unseparated after applying 3D-DSF. The extraction results are significantly different from Figure 2b. The wind may cause a relatively large offset between the ground points at different frames, indicating that past information may not provide a good reference for background filtering. Under windy weather, the point density of the cubes containing some ground points may not meet the predefined threshold. As a result, the ground points may be identified as non-background points.

The errors of background filtering under windy weather usually occur on the ground surface, because the ground surface on the road is usually smooth, and the distance between two ground circles is larger than other objects [36]. As a result, a small disturbance in the position of the LiDAR may lead to a larger offset in the location of ground surfaces. The offset in the ground surface may then cause a reduced point density in the cubes representing the ground surface, and it may increase the point density in the nearby non-background cubes. Therefore, the emphasis is on improving the accuracy of background filtering under windy weather in order to find a method to exclude the ground points effectively. This paper develops a ground surface-enhanced density statistic filtering method (GS-DSF) for background filtering. The details of the GS-DSF are documented as follows.

**Figure 3:**
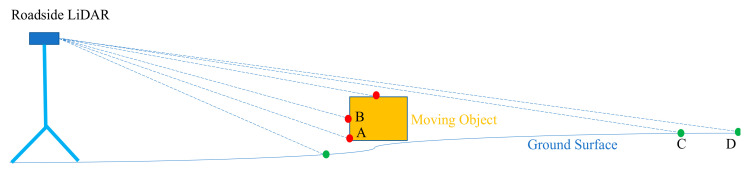
Slope difference created by moving object and ground surface.

The idea of ground surface exclusion is inspired by the ground surface exclusion used for on-board LiDAR serving autonomous vehicles [36]. The rotating LiDAR generates different circles for ground points with different distances from the LiDAR. When there is an object in the space, the slope created by the object points between two adjacent frames significantly differs from the slope created by the ground points, as shown in Figure 3.

It is shown that when a moving object appears, the slope created by the points in the moving object in two adjacent frames is much steeper than the slope created by the points in the ground surface. Equation (1) further illustrates the example in Figure 3.

(1) $$\left(\sin\left(\alpha \right)=\frac{\mathrm{Sqrt}({\left(X_{A}-X_{B}\right)}^{2}+{\left(Y_{A}-Y_{B}\right)}^{2}+{\left(Z_{A}-Z_{B}\right)}^{2})}{Z_{A}-Z_{B}}\right)>>\;(\sin\left(\beta \right)=\frac{\mathrm{Sqrt}({\left(X_{C}-X_{D}\right)}^{2}+{\left(Y_{C}-Y_{D}\right)}^{2}+{\left(Z_{C}-Z_{D}\right)}^{2})}{Z_{C}-Z_{D}})$$

where sin (α) and sin (β) represent the slopes of the moving object and the ground surface, A and B represent two points in the moving object, and C and D represent two points on the ground surface. X, Y, and Z are the XYZ coordinates (location in space) of the point. The previous study [37] found that α was usually less than 30 degrees and β was usually close to 90 degrees. In this research, we used 45 degrees as a threshold to distinguish background points and non-background points, which is named the slope-based method [37]. Since the computational load of directly applying the slope-based method on the raw LiDAR data was heavy, this paper firstly applies density statistic filtering (DSF) on the raw LiDAR data and then uses the slope-based method to exclude the ground points after DSF. The GS-DSF used here is an updated version of the traditional 3D-DSF. As mentioned before, a limitation of 3D-DSF is that the background points could not be effectively excluded under windy weather. The GS-DSF used here fixes this issue with the following updates.

The first improvement made by the GS-DSF used here is to randomly pick up the frames instead of using continuous frames. For each selected frame, the frame identity (ID) is stored (a larger ID means the frame is picked up later). The random selection can reduce the probability of picking up the frames with moving objects captured in the space. The second update of the GS-DSF which is used here is that the neighbor information is applied for background filtering. The updated GS-DSF picks up point A with the frame with the smallest ID (initial frame). Then, the neighbor of point A in other frames (except the initial frame) within a predefined distance (D) can be obtained. D is determined by the horizontal and vertical resolution. Assuming there are N randomly selected frames and n number of neighbors of point A, then the following criteria can be applied:

(2) $$\left\{\begin{array}{c} A\;is\;a\;background\;point,\;\;if\;n=N\\ A\;requires\;further\;investigation,\;\;if\;n<N \end{array}\right.$$

**Figure 4:**
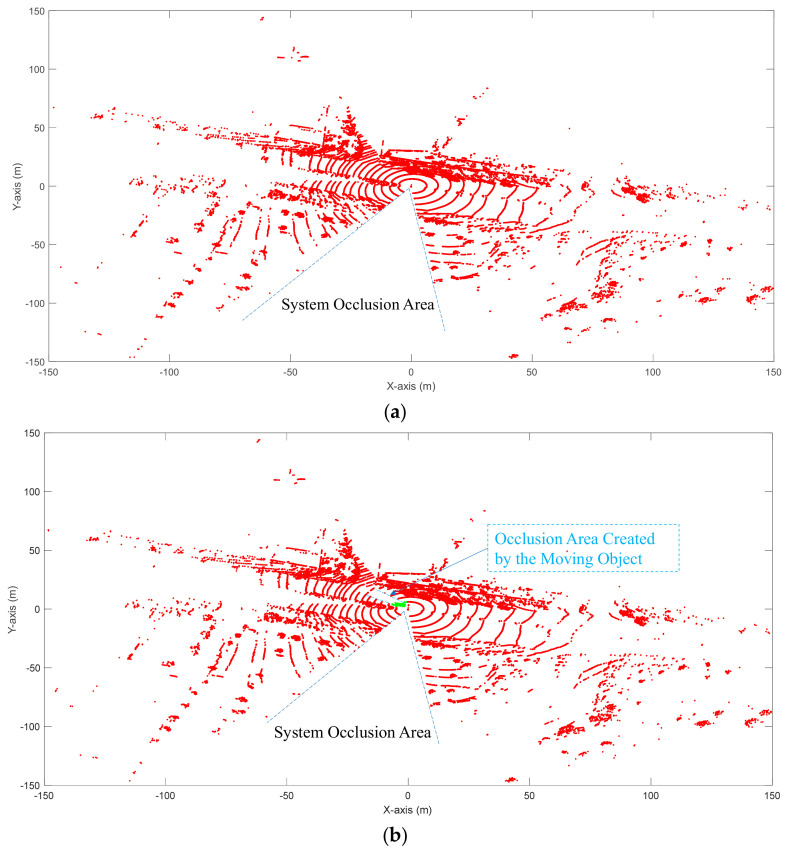
Occlusion issue: (**a**) Non-occlusion area created by moving objects, (**b**) Occlusion area created by moving objects.

If n = N, this means that point A appears in each frame in the investigated frames, indicating A is a background point. If n < N, there are two possible reasons. The first possible reason is that point A is a background point if it is blocked by the moving object in some frames. The second possible reason is that point A is a non-background point. When a moving object shows up, a vector-like blocked area is created, as shown in Figure 4.

Both Figure 4a,b have an occlusion area named the “system occlusion area”. This area was produced by the background points (such as wire pole) blocking the LiDAR. This area is invisible. As for Figure 4b, there is an occlusion area created by the moving vehicle. This occlusion area does not exist in Figure 4a. It can be clearly shown that for the occluded area, the slope between the two adjacent frames should be less than the slope created by the moving object (the same trend between α and β in Figure 3).

**Figure 5:**
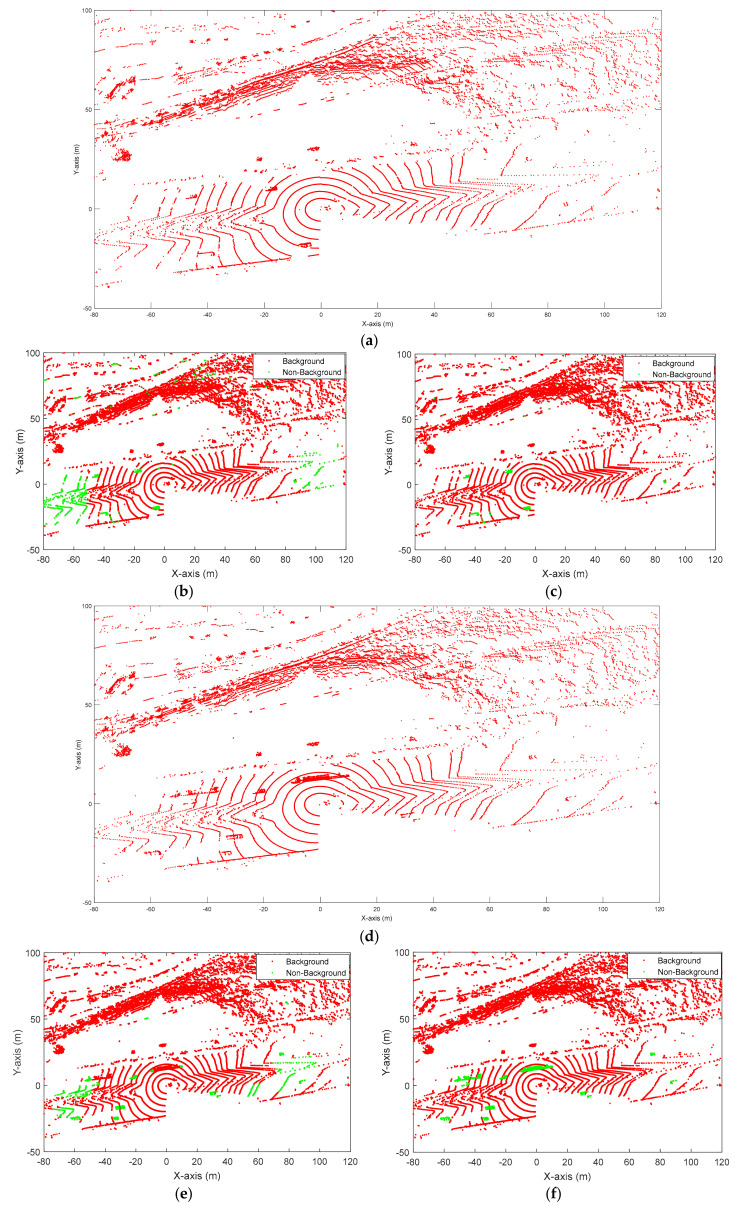
Performance of 3D-DSF and ground surface-enhanced density statistic filtering method (GS-DSF) under windy weather: (**a**) Free-flow: Raw light detection and ranging (LiDAR) data before background filtering, (**b**) Free-flow: 3D-DSF, (**c**) Free-flow: GS-DSF, (**d**) Congested traffic: Raw LiDAR data before background filtering, (**e**) Congested traffic: 3D-DSF, (**f**) Congested traffic: GS-DSF.

If n < N, it means that point A did not show up in some frames. Assuming point A did not show up in frame i, then all the points that did not show up in frame i were extracted. The slope between the two adjacent frames can then be calculated. If the average slope was shorter than 45 degrees, those points were identified as background. Otherwise, they were identified as non-background points. Figure 5 shows the results of background filtering using GS-DSF and 3D-DSF under windy weather.

It is shown that the performance of GS-DSF is better than 3D-DSF under windy weather in both free-flow and congested situations. The 3D-DSF left a lot of ground points after background filtering. When the traffic was congested, the 3D-DSF misidentified the truck which had stopped on the road as a background point. As for GS-DSF, it could exclude the background points and correctly identify the vehicle which had temporarily stopped on the road as a non-background point. To quantitatively evaluate the performance of GS-DSF, 20 frames were randomly selected under windy weather in free-flow situations and another 20 frames were randomly selected under windy weather in congested situations. Table 1 shows an example of the performance of GS-DSF and 3D-DSF (one frame in a free-flow situation and one frame in a congested situation).

The Type 1 error in Table 1 indicates the acceptance of background points as non-background points and the Type 2 error indicates the acceptance of non-background points as background points. These two types of errors can be represented as:

(3) $$\left\{\begin{array}{c} Type\;1\;error=\frac{BPF}{BP}\times 100\%\\ Type\;2\;error=\frac{VP-VPF}{VP}\times 100\% \end{array}\right.$$

It is clearly shown that both Type 1 and Type 2 errors remain low for GS-DSF under free-flow and congested situations. The two types of errors for 3D-DSF are much higher compared to GS-DSF. The Type 2 error even reached 87.2% under congested situations for 3D-DSF, indicating that a large proportion of vehicle points were misidentified as background points and were excluded from the database. The average Type 1 error and Type 2 error of GS-DSF are 0.013% and 0.642% for free-flow situations and congested situations, respectively. The average Type 1 error and Type 2 error of 3D-DSF are 0.633% and 50.614% for free-flow situations and congested situations, respectively.

**Figure 6:**
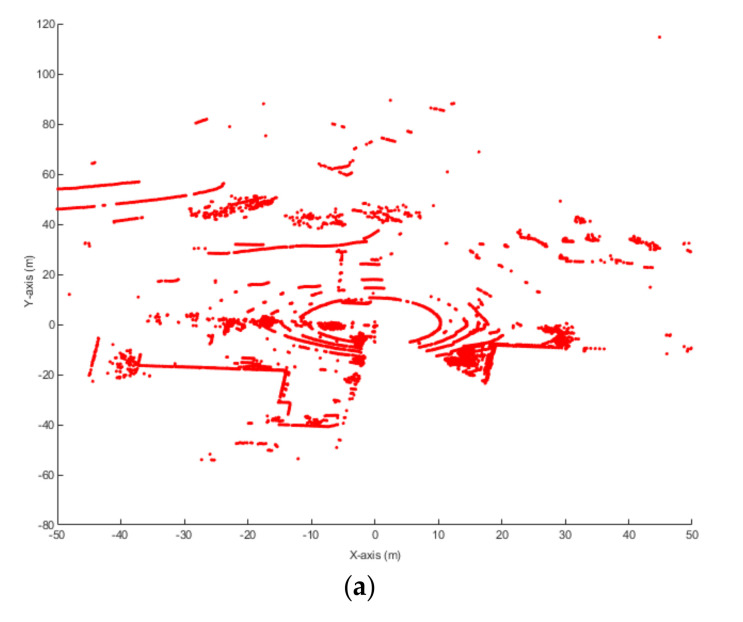

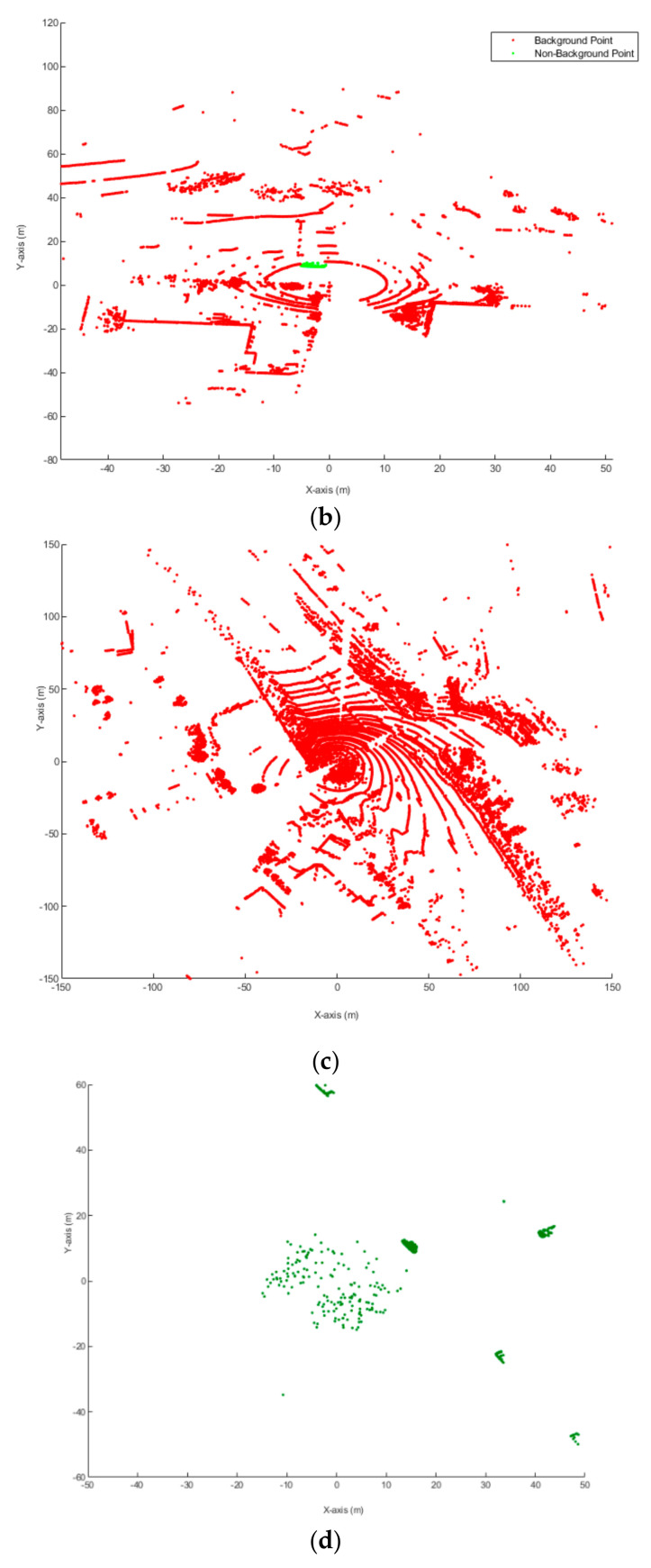
GS-DSF under rainy and snowy weather: (**a**) Rainy weather: Before GS-DSF, (**b**) Rainy weather: After GS-DSF, (**c**) Snowy weather: Before GS-DSF, (**d**) Snowy weather: After GS-DSF.

Figure 6 shows an example of GS-DSF background filtering under rainy and snowy weather.

It is shown that water drops (not under heavy rain) are invisible in the LiDAR sensors. The LiDAR points behind the water drops were blocked, leading to discontinuous ground circles and an incomplete vehicle shape, as the vehicle shape overlapped with the ground circles, as shown in Figure 6a. Under rainy weather, GS-DSF can successfully distinguish background points and non-background points, and the extracted vehicle shape is shown in Figure 6b in green. When the weather is snowy, a lot of snowflakes showed up in the LiDAR data (small dots in Figure 6c). Due to the free fall of the snowflakes, the positions of the snowflakes change in different frames. As a result, GS-DSF could not exclude the snowflakes effectively during the background filtering step (sparse dots in the center), as shown in Figure 6d. Therefore, snowflake exclusion needs to be performed in the following steps.

## 3. Point Clustering

The purpose of point clustering is to cluster the points belonging to one object into the same group. As for the roadside LiDAR data, several researchers have applied the DBSCAN-related algorithms for point clustering [32,33]. Since DBSCAN purely uses the distribution of point density as the threshold for clustering, when there are snowflakes in the space and if the snowflakes are around the object, it is possible that the snowflakes can be degree-clustered as the points object. If the mis-clustered snowflake is the point close to the roadside LiDAR (corner point), then the calculation of the speed and location of the object is inaccurate [38]. The other widely used k-means method requires an initial estimate of the number of clusters in the dataset [39]. Other researchers have used height information to cluster the LiDAR points in a space [40], but the random locations of the snowflakes can lead to false clustering results using the height-based method. Another limitation of the existing method is the heavy computational load, caused by the traversal search. Therefore, these existing methods could not meet the point clustering task under windy weather. This paper develops a fast and efficient method for point clustering. Instead of searching the point directly, this paper uses a voxelization-based method to process the data. The core of the voxelization-based method is to convert the LiDAR point into a volumetric space. The whole space is firstly divided into small cubes. Each cube can be identified as “an occupied cube” or “a non-occupied cube”. The key challenge here is how to find a reasonable side length for the cube and how to find a threshold to distinguish the occupied cube and the non-occupied cube.

**Figure 7:**
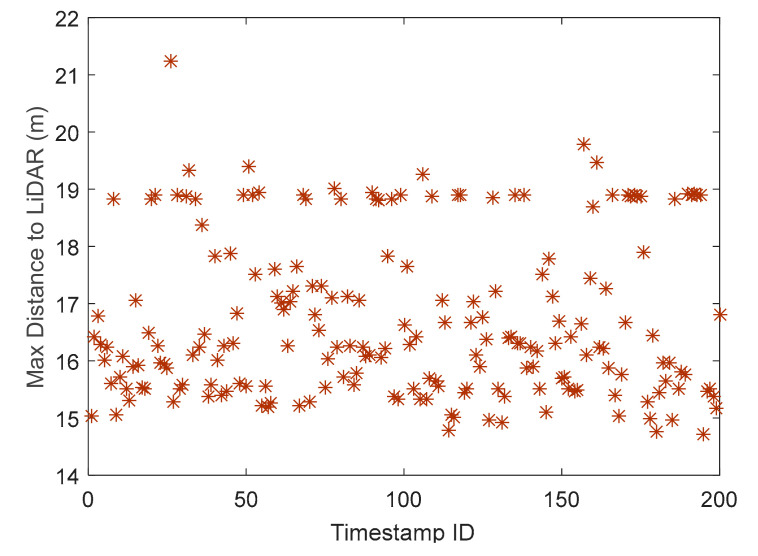
Maximum distance distribution of the snowflakes to the LiDAR.

The point distribution feature of the snowflakes was firstly analyzed. About 10 h of LiDAR data under heavy snow weather were collected. A total of 200 frames were randomly selected for investigation. The maximum distance of the snowflakes among the 200 frames is shown in Figure 7.

It is shown that the maximum distance of the snowflake is less than 22 m in all frames. When the distance is longer than 22 m, the reflection of the snowflake is too weak to be detected by the LiDAR. This feature indicates that the influence range of the snowflakes on the data is limited to 22 m from the LiDAR. The reason for this phenomenon is that the snowflakes can scatter the laser and reduce the intensity of the reflection.

The LiDAR measures the reflectivity of an object with 256-bit resolution, independent of laser power and distance over a range from 1 m to 100 m. Commercially available reflectivity standards and retro-reflectors are used for the absolute calibration of the reflectivity.

- Diffuse reflectors report values from 0–100 for the range of reflectivity from 0% to 100%.
- Retro-reflectors report values from 101 to 255 with 255 being the reported reflectivity for an ideal retro-reflector and 101–254 being the reported reflectivity for partially obstructed or imperfect retro-reflectors.

**Figure 8:**
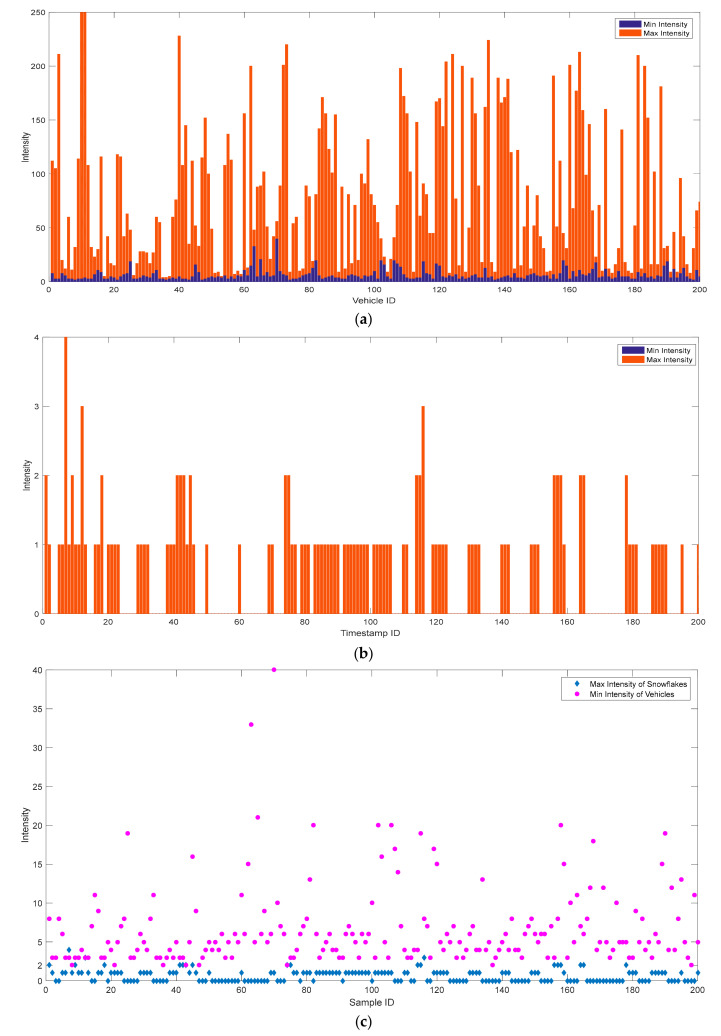
Intensity features of vehicles and snowflakes: (**a**) Intensity of vehicles, (**b**) Intensity of snowflakes, (**c**) Comparison of maximum intensity of snowflakes and minimum intensity of vehicles.

The distribution of intensity of the snowflakes and the vehicles is shown in Figure 8.

**Figure 9:**
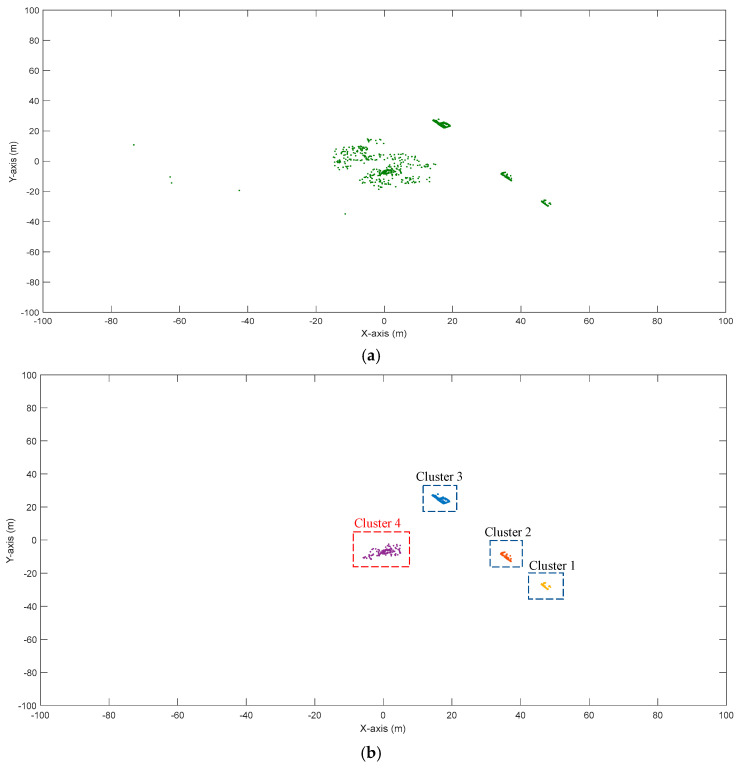

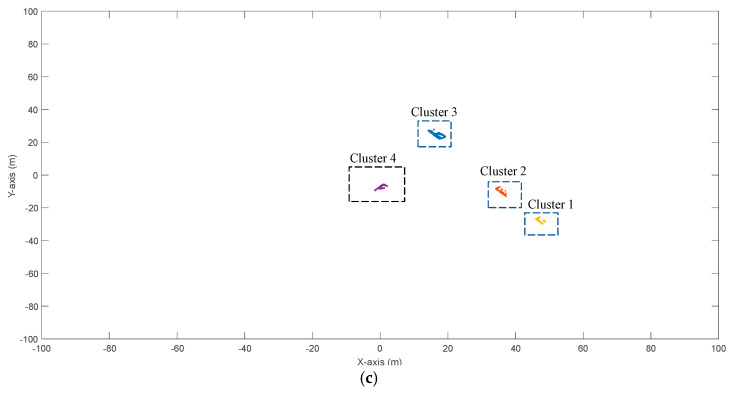
Point clustering: (**a**) Before point clustering, (**b**) Revised density-based spatial clustering of applications with noise (DBSCAN), (**c**) Proposed method.

It can be seen that the maximum intensity of the vehicles varied in a larger range compared to that of the snowflakes. The absolute value of the maximum intensity of the vehicles is also larger than that of the snowflakes. Therefore, to better distinguish vehicles and snowflakes, we used the minimum intensity of vehicles. Then, the comparison of the maximum intensity of the snowflakes and the minimum intensity of the vehicles showed that the maximum intensity of most snowflakes was less than the minimum intensity of the vehicles, which suggested that the two indexes could help distinguish vehicles and snowflakes. By analyzing 100 randomly selected frames, it was also found that 98.5% of snowflakes had a maximum intensity of less than two and 96% of vehicles had a minimum intensity larger than two. The minimum intensity of the snowflakes was zero, indicating that the LiDAR did not receive the signal that it sent out. As for the snowflakes, the minimum intensity was zero and the maximum intensity was two (for 98.5%), but for the vehicles, the minimum intensity was usually more than two. Therefore, the value of two was selected as a threshold to distinguish the snowflakes and vehicles. The points with a minimum intensity higher than two were considered as non-snowflakes and the points with a maximum intensity less than two were considered as snowflakes and were removed from the space. For the points with an intensity equal to two, they were left in the space and clustered based on the revised DBSCAN algorithm proposed by Zhao et al. [19]. Figure 9 shows the point clustering with the proposed method and the revised DBSCAN algorithm developed in [19]. A cluster refers to points that can be categorized into one group. It can be seen that there were no obvious differences in Clusters 1–3 using the two methods. The influence of the snowflakes only occurred within 20 m of the LiDAR [20]. Therefore, only Cluster 4 was different under the two methods. For Cluster 4, the revised DBSCAN algorithm mis-clustered a lot of snowflakes around the vehicle as vehicle points while the proposed algorithm successfully excludes snowflakes and keeps the vehicle points in the space.

To further evaluate the performance of the proposed method, the proposed method and the methods developed in [5] and [19] were used to process the same LiDAR databases collected in windy weather and snowy weather (125 and 651 data for each scenario, respectively). Table 2 summarizes the results of the three methods. Though there were still some errors in counting the vehicle volume under both snowy and windy weather using the proposed method, the accuracy was greatly improved compared to the methods in [5] and [19]. An overall accuracy of more than 90% can be achieved with the proposed method. The evaluation shows that the performance of the proposed method is superior compared to the state-of-the-art methods.

## 4. Conclusions and Discussion

This paper evaluates the performance of the state-of-the-art methods of background filtering and point clustering for roadside LiDAR data under windy and snowy weather. The results showed that the existing background filtering and point clustering methods could not process the roadside LiDAR data effectively. This paper develops a ground surface-enhanced point density statistics filtering method to exclude the background points under windy weather. The intensity information was used to improve the accuracy of the revised DBSCAN algorithm developed by Zhao et al. [19]. The testing results showed that the proposed methods can exclude the background points and cluster the vehicle points into one group effectively under windy and snowy weather.

There are already some algorithms developed for autonomous vehicles, such as those in [23]. However, those algorithms serving for autonomous vehicles could not be directly applied to the connected vehicles since the working environment and region of interest are different. There are still some limitations that can be improved in the future. Foggy weather can also significantly decrease the quality of the LiDAR data. However, LiDAR data under foggy weather was not available for this research. Future studies should evaluate the performance of the proposed methods using the LiDAR data under foggy and smoggy weather. This paper manually selects two as the intensity value to identify the snowflakes, but a more advanced method to automatically select the threshold is still needed.

## Figures and Tables

**Table 1:** Quantitative Evaluation of ground surface-enhanced density statistic filtering (GS-DSF) and 3D density statistic filtering (3D-DSF)**.**

	Background Points (BP)	Vehicle Points (VP)	Background Points after Filtering (BPF)		Vehicles Points after Filtering (VPF)		Type 1 Error	Type 2 Error
Free-Flow	598,512	9873	GS-DSF	59	GS-DSF	9789	0.0098%	0.8508%
			3D-DSF	3615	3D-DSF	9802	0.6040%	0.7191%
Congested Situation	599,982	20,172	GS-DSF	71	GS-DSF	20,150	0.0118%	0.1091%
			3D-DSF	3429	3D-DSF	2578	0.5715%	87.2199%

**Table 2:** Performance evaluation.

	Snowy Weather			Windy Weather		
Actual Number of Vehicles	125			651		
Methods	Proposed Method	Method in [5]	Method in [19]	Proposed Method	Method in [5]	Method in [19]
Detected number of vehicles	135	190	145	689	781	725
Error (%)	8.0	52.0	16.0	5.8	19.9	11.4
